# WWP1 knockout in mice exacerbates obesity‐related phenotypes in white adipose tissue but improves whole‐body glucose metabolism

**DOI:** 10.1002/2211-5463.12795

**Published:** 2020-02-03

**Authors:** Shunsuke Hoshino, Masaki Kobayashi, Ryoma Tagawa, Ryutaro Konno, Takuro Abe, Kazuhiro Furuya, Kumi Miura, Hiroki Wakasawa, Naoyuki Okita, Yuka Sudo, Yuhei Mizunoe, Yoshimi Nakagawa, Takeshi Nakamura, Hiroshi Kawabe, Yoshikazu Higami

**Affiliations:** ^1^ Laboratory of Molecular Pathology and Metabolic Disease Faculty of Pharmaceutical Sciences Tokyo University of Science Noda Japan; ^2^ Division of Pathological Biochemistry Faculty of Pharmaceutical Sciences Sanyo‐Onoda City University Sanyo‐onoda Japan; ^3^ Department of Internal Medicine (Endocrinology and Metabolism) Faculty of Medicine University of Tsukuba Tsukuba Japan; ^4^ Division of Biosignaling Research Institute for Biomedical Sciences Tokyo University of Science Noda Japan; ^5^ Department of Molecular Neurobiology Max Planck Institute of Experimental Medicine Göttingen Germany; ^6^ Division of Pathogenic Signaling Department of Biochemistry and Molecular Biology Kobe University Graduate School of Medicine Chuo‐ku, Kobe Japan; ^7^ Department of Gerontology Laboratory of Molecular Life Science Institute of Biomedical Research and Innovation Foundation for Biomedical Research and Innovation at Kobe Japan

**Keywords:** white adipose tissue, obesity, WWP1, antioxidative capacity, mitochondrial function

## Abstract

White adipose tissue (WAT) is important for maintenance of homeostasis, because it stores energy and secretes adipokines. The WAT of obese people demonstrates mitochondrial dysfunction, accompanied by oxidative stress, which leads to insulin resistance. WW domain‐containing E3 ubiquitin protein ligase 1 (WWP1) is a member of the HECT‐type E3 family of ubiquitin ligases and is associated with several diseases. Recently, we demonstrated that WWP1 is induced specifically in the WAT of obese mice, where it protects against oxidative stress. Here, we investigated the function of WWP1 in WAT of obese mice by analyzing the phenotype of *Wwp1* knockout (KO) mice fed a high‐fat diet. The levels of oxidative stress markers were higher in obese WAT from *Wwp1* KO mice. Moreover, *Wwp1* KO mice had lower activity of citrate synthase, a mitochondrial enzyme. We also measured AKT phosphorylation in obese WAT and found lower levels in *Wwp1* KO mice. However, plasma insulin level was low and glucose level was unchanged in obese *Wwp1* KO mice. Moreover, both glucose tolerance test and insulin tolerance test were improved in obese *Wwp1* KO mice. These findings indicate that WWP1 participates in the antioxidative response and mitochondrial function in WAT, but knockdown of WWP1 improves whole‐body glucose metabolism.

## Graphical abstract

WWP1 knockout mice fed high‐fat diet have suppressed mitochondrial enzymatic activity and exacerbated oxidative stress in white adipose tissue, but decreased blood insulin levels and enhanced insulin sensitivity, resulting in improved glucose metabolism in whole body.

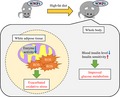

**Abbreviations**
4‐HNE: 4‐hydroxy‐2‐nonenal
CBB: Coomassie Brilliant Blue
CoxII: cytochrome c oxidase subunit II
CS: citrate synthase
DTNB: 5,5‐dithio‐bis‐(2‐nitrobenzoic) acid
GTT: glucose tolerance test
HFD: high‐fat diet
Hprt: hypoxanthine guanine phosphoribosyl transferase
ITT: insulin tolerance test
KLF2: Kruppel‐like factor 2
KLF5: Kruppel‐like factor 5
KO: knockout
mtDNA: mitochondrial DNA
ND: normal diet
OCR: oxygen consumption rate
Pparγ: peroxisome proliferator‐activated receptor γ
PTEN: phosphatase and tensin homolog
ROS: reactive oxygen species
Rps18: ribosomal protein S18
Sod1: superoxide dismutase 1
Sod2: superoxide dismutase 2
SPG20: spastic paraplegia 20
TCA cycle: tricarboxylic acid cycle
TGFβ: transforming growth factor β
TNB: thio‐bis‐(2‐nitrobenzoic) acid
Trx1: thioredoxin 1
Trx2: thioredoxin 2
WAT: white adipose tissue
WT: wild‐type
WWP1: WW domain‐containing E3 ubiquitin protein ligase 1

White adipose tissue (WAT) is important for maintenance of homeostasis, because it stores energy in the form of triglyceride and secretes adipokines that affect energy metabolism by modulating the activities of adiponectin, leptin, and proinflammatory cytokines. The main component of WAT is white adipocytes containing unilocular lipid droplets that hypertrophy and store excess lipid under conditions of obesity. Hypertrophy of mature adipocytes disrupts the balance in adipokine secretion, as it is accompanied by reduction in adiponectin secretion and increased release of proinflammatory cytokines that induce systemic insulin resistance, chronic inflammation, and oxidative stress 1, 2.

Reactive oxygen species (ROS) are generated primarily in mitochondria and affect numerous signaling pathways 3, 4, 5. Generally, oxidative stress is caused when the quantity of ROS generated exceeds the capacity of the antioxidant defense systems, which can result in inflammation and cell death 6, 7. Higher concentrations of ROS in WAT are closely associated with obesity‐related pathologies such as hypertension, type 2 diabetes, and dyslipidemia 8, 9. Mitochondrial enzymes, which include those of the tricarboxylic acid cycle (TCA cycle), the β‐oxidation process, and the electron transfer system, are important for both aerobic ATP synthesis and production of ROS. Mitochondrial dysfunction disrupts aerobic metabolism, which increases ROS production and impairs energy homeostasis. Mitochondria in WAT of obese people produce more ROS and express increased levels of markers of oxidative damage 10. These observations indicate that oxidative stress because of increased mitochondrial ROS contributes to obesity‐related pathologies.

WW domain‐containing E3 ubiquitin protein ligase 1 (WWP1), also known as TIUL1 or AIP5, belongs to the HECT‐type E3 ubiquitin ligase family. WWP1 has a C2 domain at its N‐terminal and four WW domains in its central region 11, 12. The C2 domain determines subcellular localization of the molecule, while the WW domains bind to proline‐rich sequences (PY motif) of substrate proteins. WWP1 has been reported to be associated with various diseases. For example, WWP1 is highly expressed in high‐grade breast cancer with poor prognosis 13, whereas low expression of WWP1 protein is associated with sarcopenia and abnormal neurogenesis 14, 15, 16. Therefore, it is likely that the pathophysiologic roles of WWP1 differ between normal and cancerous tissues.

The substrates of WWP1 include Kruppel‐like factor 2 (KLF2) 17, KLF5 18, 19, and spastic paraplegia 20 (SPG20) 20, 21. It is known that KLF2 and KLF5 are involved in adipocyte differentiation 18, 22, while SPG20 regulates the size and number of lipid droplets 20. Therefore, it is likely that WWP1 is involved in determining the phenotype of adipocytes.

Recently, we found that WWP1 is induced specifically in obese mouse WAT and is protective against oxidative stress in adipocytes 23. However, we did not provide *in vivo* evidence for the roles of WWP1 in WAT. Therefore, in the present study, we explored the function of WWP1 in obese WAT and whole‐body glucose metabolism by analyzing the phenotype of obese *Wwp1* knockout (KO) mice.

## Materials and methods

### Animals

Animal experiments were approved by the Ethics Review Committee for Animal Experimentation at Tokyo University of Science (approval number: Y19054). Mice with global KO of *WWp1* (*Wwp1* ^−/−^ mice) and wild‐type (WT) *Wwp1* ^+/+^ mice were generated by mating *Wwp1* ^fl/fl^/*Wwp2* ^+/+^ (*Wwp1* ^fl/fl^) mice, which were obtained by crossing *Wwp1* ^fl/+^/*Wwp2* ^fl/+^ mice with CAG‐Cre mice. *Wwp1* ^fl/fl^/*Wwp2* ^fl/fl^ mice were provided by Dr. Hiroshi Kawabe (Max Planck Institute of Experimental Medicine, Göttingen, Germany) 16, and CAG‐Cre mice were kindly provided by Dr. Takeshi Nakamura (Research Institute for Biomedical Sciences, Tokyo University of Science, Chiba, Japan). *Wwp1* ^fl/+^/*Wwp2* ^fl/+^ mice were obtained by crossing *Wwp1* ^fl/fl^/*Wwp2* ^fl/fl^ and C57/BL6 mice. Genotyping of offspring was performed by PCR using KOD FX neo (Toyobo, Osaka, Japan) or Quick Taq^®^ HS DyeMix (Toyobo). Primers used for genotyping were as follows: *Wwp1* ^fl/fl^ (forward, 5′‐AGA GGC AAG AGA ATG GCG TCA AG‐3′; reverse, 5′‐CAG AAA CAG TGA TCT ACA AGT CTA AAT G‐3′), *Wwp2* ^fl/fl^ (forward, 5′‐GAT GGT TGT GAG CCA CTT ACT TC‐3′; reverse, 5′‐CCA TCA TGA ACT TCA GCC TC‐3′), *Wwp1* ^−/+^ (forward, 5′‐AGA GGC AAG AGA ATG GCG TCA AG‐3′; reverse, 5′‐GGA GGT GAA AGG GTT GGA AGA ATA C‐3′), and CAG‐Cre (forward, 5′‐ACC TGA AGA TGT TCG CGA TTA TCT‐3′; reverse, 5′‐ACC GTC AGT ACG TGA GAT ATC TT‐3′).

Mice were maintained under specific‐pathogen‐free conditions at 23°C, under a 12‐h light/dark cycle in the animal facility at the Faculty of Pharmaceutical Sciences, Tokyo University of Science. They had free access to water and were fed a Charles River Formula‐1 diet (21.9% crude protein, 5.4% crude fat, and 2.9% crude fiber; Oriental Yeast, Japan). At 5 weeks old, WT and KO mice were allocated to two groups: the normal diet (ND) group or high‐fat diet (HFD) group. Labo MR Stock and High‐Fat Diet 32 (25.5% crude protein, 32.0% crude lipid, and 2.9% crude fiber; CREA, Tokyo, Japan) were fed as the ND and HFD, respectively. At 13 weeks old, mice were euthanized under isoflurane anesthesia (Mylan, Canonsburg, PA, USA) in a fed state, and their epididymal WAT depots and liver were collected and weighed. These tissues were immediately diced, frozen in liquid nitrogen, and stored at − 80°C. Blood samples were mixed with 100 mm EDTA in 1.5‐mL microtubes and centrifuged at 2,500 × *g* for 10 min at 4°C, and plasma was collected and stored at −80°C until analysis.

### Cell lines

3T3‐L1 preadipocytes were purchased from RIKEN Bioresource Center (Ibaraki, Japan). 3T3‐L1/shGFP and 3T3‐L1/shWwp1 preadipocytes were previously established in our laboratory using a retrovirus system 23.

### Cell culture and differentiation

3T3‐L1 preadipocytes were maintained in Dulbecco’s modified Eagle’s medium (low glucose) (Wako, Osaka, Japan) containing 10% fetal bovine serum (Thermo; Waltham, MA, USA) and 1% penicillin/streptomycin (Millipore Sigma, St. Louis, MO, USA). Differentiation of 3T3‐L1 preadipocytes to adipocytes was performed as previously described 24.

### Immunoblotting

The preparation of WAT lysates and immunoblotting was performed according to our previously reported methods 24. Briefly, WAT was lysed in SDS sample buffer (50 mm Tris‐HCl (pH 6.8), 2% SDS, 3 M urea, 6% glycerol), boiled for 5 min, and sonicated. Lysates were subjected to SDS/PAGE (15 μg protein per well), and separated proteins were transferred to nitrocellulose membranes. Membranes were blocked with blocking solution (2.5% skim milk, 0.25% BSA in TTBS) (25 mm Tris‐HCl pH 7.4, 140 mm NaCl, 2.5 mm KCl, 0.1% Tween‐20) for 1 h at room temperature and then probed with appropriate primary antibodies overnight at 4°C. The anti‐WWP1 antibody was originally generated in our laboratory 23, anti‐phospho‐Akt (Ser473; #9271) and anti‐Akt antibodies (#9272) were from Cell Signaling Technology (Danvers, MA, USA), and the anti‐4‐hydroxy‐2‐nonenal (4‐HNE) antibody was from JaICA (MHN‐020P; Shizuoka, Japan). Since the anti‐4‐HNE antibody nonspecifically recognizes 4‐HNE‐modified proteins, 4‐HNE intensity was analyzed over the entire area per well on an immunoblotting membrane. Subsequently, membranes were incubated with appropriate secondary antibodies for 1 h at room temperature [horseradish peroxidase‐conjugated F(ab’)2 fragment of goat anti‐mouse IgG or anti‐rabbit IgG (Jackson Immuno Research, West Grove, PA, USA)]. Antibody‐bound proteins were visualized using ImmunoStar LD Reagent (Wako) and an LAS3000 Image Analyzer (Fujifilm, Tokyo, Japan), and data were analyzed using multigauge software (GE Healthcare, Madison, WI, USA). The intensity of Coomassie Brilliant Blue (CBB) staining of membrane proteins was used to normalize target protein expression levels.

### Quantitative real‐time RT‐PCR

Total RNA was extracted from frozen WAT using ISOGEN II (Nippon gene, Toyama, Japan), and reverse transcription was performed using ReverTra Ace® qPCR RT Master Mix (Toyobo). Quantitative real‐time PCR was performed using the CFX Connect^TM^ Real Time System (Bio‐Rad, Hercules, CA, USA) and Thunderbird SYBR qPCR mix (Toyobo), according to manufacturer protocols. Sequences of primers used for PCR are as follows: *Adiponectin (Adipoq)* (forward, 5′‐TGC CGA AGA TGA CGT TAC TAC AAC‐3′; reverse, 5′‐CTT CAG CTC CTG TCA TTC CAA C‐3′), *Catalase* (forward, 5′‐CCA AGG CAA AGG TGT TTG AG‐3′; reverse, 5′‐GGG TTT CTC TTC TGG CTA TGG‐3′), *Leptin* (forward, 5′‐CCA GGA TCA ATG ACA TTT CAC ACA C‐3′; reverse, 5′‐CAG GGA GCA GCT CTT GGA GAA G‐3′), *Peroxiredoxin1 (Prdx1)* (forward, 5′‐GCT TTC AGT GAT AGA GCC GAT G‐3′; reverse, 5′‐CCA TAA TCC TGA GCA ATG GTG‐3′), *Peroxisome Proliferator‐Activated Receptor γ* (*Pparγ)* (forward, 5′‐CAC AAT GCC ATC AGG TTT GG‐3′; reverse, 5′‐GCG GGA AGG ACT TTA TGT ATG AG‐3′), *Ribosomal Protein S18* (*Rps18)* (forward, 5′‐TGC GAG TAC TCA ACA CCA ACA T‐3′; reverse, 5′‐CTT TCC TCA ACA CCA CAT GAG C‐3′), *Superoxide dismutase 1* (*Sod1)* (forward, 5′‐GGA TGA AGA GAG GCA TGT TGG‐3′; reverse, 5′‐TTT GCC CAA GTC ATC TTG TTT C‐3′), *Superoxide dismutase 2 (Sod2)* (forward, 5′‐CCC AAA GGA GAG TTG CTG GAG‐3′; reverse, 5′‐CGA CCT TGC TCC TTA TTG AAG C‐3′), *Thioredoxin1 (Trx1)* (forward, 5′‐AGC TGA TCG AGA GCA AGG AAG‐3′; reverse, 5′‐ATT TTG CAA GGT CCA CAC CAC‐3′), and *Thioredoxin2 (Trx2)* (forward, 5′‐TTC ATG CAC AGT GGT GTG G‐3′; reverse, 5′‐TCA ATG GCA AGG TCT GTG TG‐3′). *Rps18* was used as a housekeeping gene.

### Plasma biochemical analyses

Plasma glucose, insulin, and leptin levels were measured using Autokit Glucose (Wako), a Mouse Insulin ELISA KIT (Utype) (Shibayagi, Japan), and a Quantikine^®^ ELISA Mouse/Rat Leptin Immunoassay (R&D Systems, Minneapolis, MN, USA), respectively. All assays were performed according to manufacturer protocols.

### Intraperitoneal glucose tolerance test and insulin tolerance test

Glucose tolerance test (GTT) and insulin tolerance test (ITT) were performed using HFD‐fed *Wwp1* WT and KO mice at 13–15 weeks old. Prior to GTT and ITT, mice were fasted for 24 h. d‐glucose (1.0 g/kg body weight, Wako) or insulin (1.0 U/mL body weight, Wako) were injected intraperitoneally for GTT or ITT, respectively. Next, serial blood sampling from the tail vein was performed at 0, 30, 60, and 120 min after injection. Blood glucose levels were measured using an Accu‐chek^®^ aviva blood glucose meter (Roche).

### Measurement of glutathione concentrations

Total glutathione (tGSH [GSH + glutathione disulfide (GSSG)]) and GSSG concentrations were measured as previously reported 25. WAT was homogenized in extraction buffer (0.1M potassium phosphate buffer containing 5 mm EDTA (pH 7.5), 0.1% Triton X‐100, and 0.6% sulfosalicylic acid) and centrifuged at 4°C for 10 min. Supernatants were used for the measurement of tissue GSH content with an Infinite F200 PRO microplate reader (Tecan; Männedorf, Switzerland). The rate of 5,5‐dithio‐bis‐(2‐nitrobenzoic) acid (DTNB) formation was calculated, and the concentrations of tGSH and GSSG in each sample were determined using linear regression, with reference to a standard curve. GSH concentration was calculated by subtracting GSSG concentration from tGSH concentration.

### Measurement of citrate synthase activity

To prepare lysates for the measurement of mitochondrial activity, WAT samples were homogenized in homogenization buffer containing 50 mm Tris‐HCl, pH 7.4, 150 mm NaCl, 1% phosphatase inhibitor cocktail (Thermo), 5 mm EDTA, 1% protease inhibitor cocktail (Sigma), 1% NP‐40, and 0.05% sodium deoxycholate. Protein concentration in each lysate was determined using a BCA protein assay kit (Thermo Scientific; IL, USA), according to the manufacturer’s protocol. Citrate synthase (CS) activity was measured by monitoring color development associated with thio‐bis‐(2‐nitrobenzoic) acid (TNB) generation from reduction of DTNB by CoA‐SH, the byproduct of citrate synthesis 24. After addition of reaction buffer, samples were incubated at 25°C for 5 min, and the reaction was initiated by addition of 0.5 mm oxaloacetate. Changes in absorbance at 412 nm were recorded for at least 3 min using an ARVO MX/Light Wallac 1420 Multilabel/Luminescence Counter (PerkinElmer; MA, USA).

### Measurement of mitochondrial DNA content

DNA was separated and subjected to quantitative PCR using primers for *CoxII* (cytochrome c oxidase subunit II), which is encoded by mitochondrial DNA (mtDNA) (forward, 5′‐CCA TCC CAG GCC GAC TAA‐3′; reverse, 5′‐AAT TTC AGA GCA TTG GCC ATA GA‐3′) or for *Hprt* (hypoxanthine guanine phosphoribosyl transferase), which is encoded by genomic DNA (forward, 5′‐ATC CAG GTT ACA AGG CAG CT‐3′; reverse, 5′‐GGG AAA CAT AGA CAG GGG‐3′). Quantitative PCR was performed using the CFX Connect^TM^ Real Time System (Bio‐Rad) and Thunderbird SYBR qPCR mix (Toyobo), according to manufacturer protocols. Relative mitochondrial copy number is represented by the amplification ratio of *CoxII* to *Hprt*.

### Measurement of oxygen consumption rate

Oxygen consumption rate (OCR) was measured as previously reported 25 in 3T3‐L1/shGFP and 3T3‐L1/shWwp1 cell lines after differentiation into mature adipocytes. The measurement was made using a Seahorse XF analyzer (Agilent Technologies, Santa Clara, CA, USA), according to the manufacturer’s protocol.

### Statistical analysis

The Tukey–Kramer test was used to analyze data, using r software (R project for Statistical Computing). Differences with *P* values < 0.05 were considered statistically significant.

## Results

Deletion of WWP1 in WAT of *Wwp1* KO mice was confirmed at the protein level (Fig. S1A). Under ND‐ and HFD‐fed conditions, *Wwp1* KO mice showed no differences in body mass, caloric intake, or WAT mass (Fig. S1B–D).

**Figure 1:**
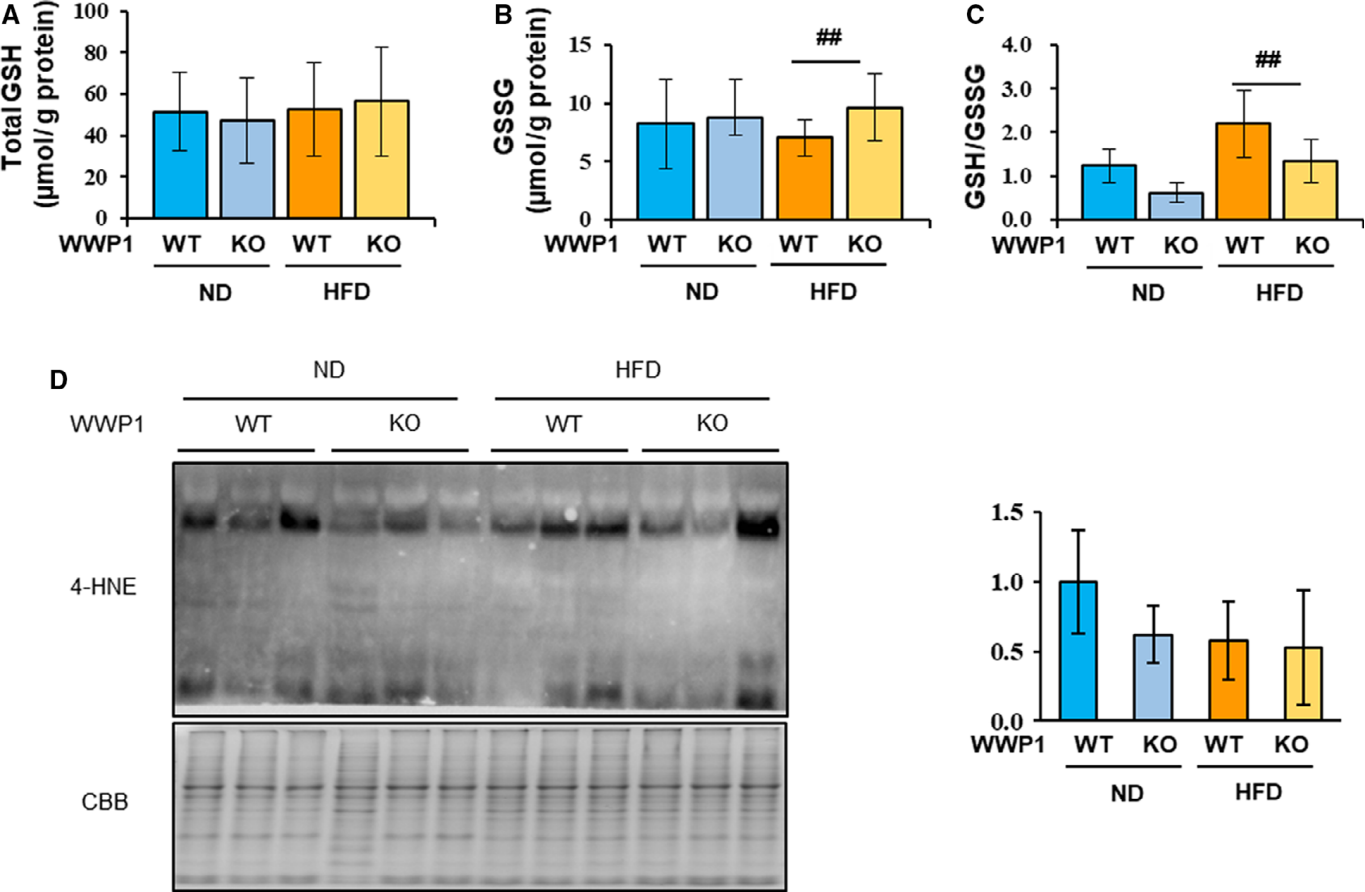
WWP1 is involved in antioxidative reactions in obese WAT. (A–C) Glutathione concentrations in WAT from all groups of mice were measured spectrophotometrically at 412 nm. (A) total GSH, (B) GSSG, (C) GSH/GSSG ratio. (D) Immunoblotting of 4‐HNE in WAT lysates from all groups of mice. CBB staining was used as the loading control. Quantitative values represent mean ± SD (*n* = 3–5 per group). Differences between these values were analyzed using the Tukey–Kramer test [##, *P* < 0.01 *Wwp1* WT mice (HFD‐fed) *vs*. *Wwp1* KO mice (HFD‐fed)].

To evaluate the levels of oxidative stress in WAT, we measured the GSH/GSSG ratio and concentration of 4‐HNE adduct, which are widely used markers of oxidative stress. HFD‐fed *Wwp1* KO mice had significantly higher GSSG concentrations and lower GSH/GSSG ratio than WT mice, and these differences were not observed in ND‐fed mice (Fig. 1A–C). However, WAT 4‐HNE adduct concentrations were similar in all groups (Fig. 1D). These results suggest that WWP1 contributes to the antioxidative response in obese WAT.

**Figure 2:**
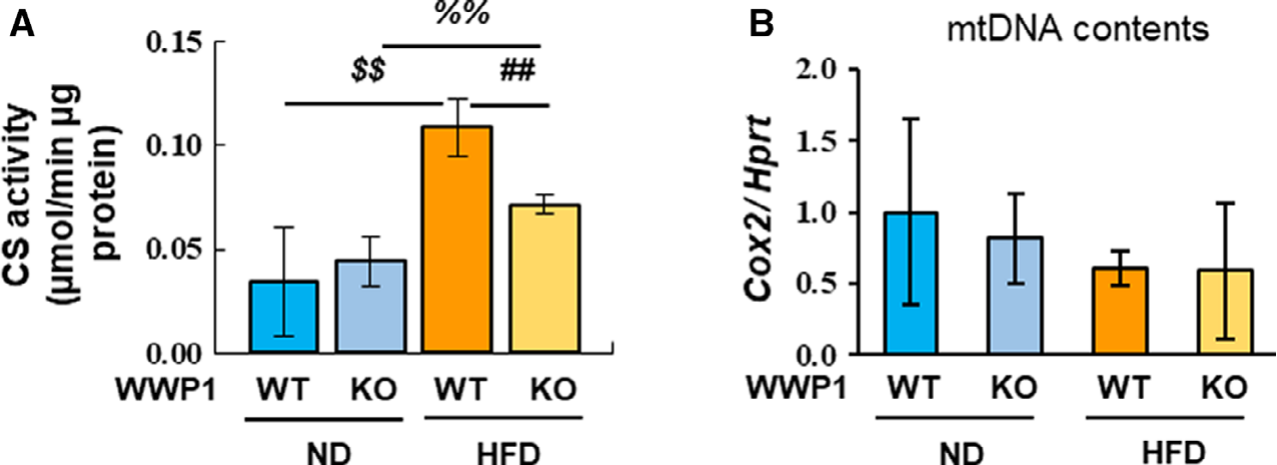
The WWP1 is required for normal mitochondrial function in obese WAT. (A) CS activity in WAT from all groups of mice was measured spectrophotometrically at 412 nm. (B) Genomic *Cox2* levels were quantified using real‐time PCR (*n* = 3–5). Quantitative values represent mean ± SD. Differences between these values were analyzed using the Tukey–Kramer test [##, *P* < 0.01 *Wwp1* WT mice (HFD‐fed) vs. *Wwp1* KO mice (HFD‐fed); $$, *P* < 0.01 *Wwp1* WT mice (ND‐fed) vs. *Wwp1* WT mice (HFD‐fed); %%, *P* < 0.01 *Wwp1* KO mice (ND‐fed) vs. *Wwp1* KO mice (HFD‐fed)].

To determine whether WWP1 affects oxidative stress by influencing mitochondrial function, we measured the enzymatic activity of CS, a rate‐limiting enzyme in the TCA cycle, and the level of mtDNA, a surrogate for mitochondrial content. We found that CS activity was higher in HFD‐fed mice and lower in *Wwp1* KO mice than in WT mice (Fig. 2A). In contrast, *Wwp1* KO mice showed no significant differences from WT mice in mtDNA levels under both ND‐ and HFD‐fed conditions (Fig. 2B). These results suggest that WWP1 may influence mitochondrial activity, but not mitochondrial mass, in WAT.

**Figure 3:**
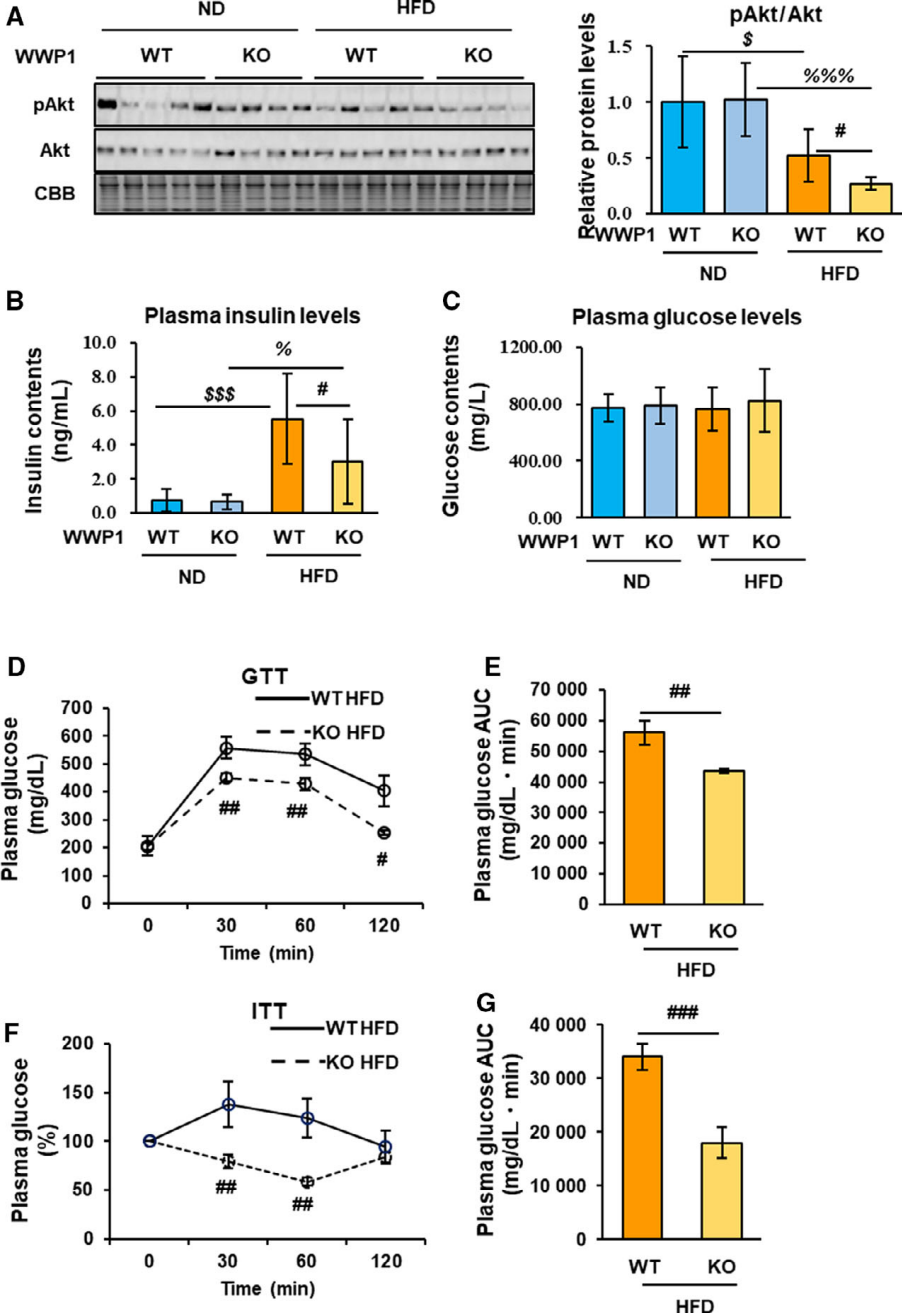
The WWP1 contributes to the maintenance of insulin sensitivity in obese WAT. (A) Immunoblotting for phosphorylated AKT (pAKT) and total AKT in WAT from all groups of mice. (B, C) Plasma insulin (B) and glucose (C) levels from all groups of mice. (D‐G) Blood glucose levels during GTT (D) or ITT (F) in HFD‐fed *Wwp1* WT and KO mice. Each area under the curve (AUC) is calculated and shown as (E) or (G). Quantitative data represent mean ± SD (*n* = 4–6 per group). Differences between these values were analyzed using the Tukey–Kramer test [#, *P* < 0.05, ##, *P* < 0.01, ###, *P* < 0.001, *Wwp1* WT mice (HFD‐fed) vs. *Wwp1* KO mice (HFD‐fed); $, *P* < 0.05, $$$, *P* < 0.001 *Wwp1* WT mice (ND‐fed) vs. *Wwp1* WT mice (HFD‐fed); %%%, *P* < 0.001 *Wwp1* KO mice (ND‐fed) vs. *Wwp1* KO mice (HFD‐fed)].

Obesity‐induced ROS accumulation attenuates insulin signal transduction in WAT 26, 27. To assess the effects of WWP1 on insulin signaling, we measured AKT phosphorylation (pAKT), a major intermediate of insulin signaling, and plasma glucose and insulin levels in WAT from *Wwp1* WT and KO mice. The results showed that HFD‐feeding induced a significant reduction in the pAKT/total AKT ratio, which was more marked in KO mice (Fig. 3A). Plasma insulin levels were decreased, but glucose levels were unchanged in KO mice (Fig. 3B,C). We performed GTT and ITT. Unexpectedly, both tests improved in KO mice, and WWP1 deficiency appeared to improve glucose metabolism (Fig. 3D–G).

**Figure 4:**
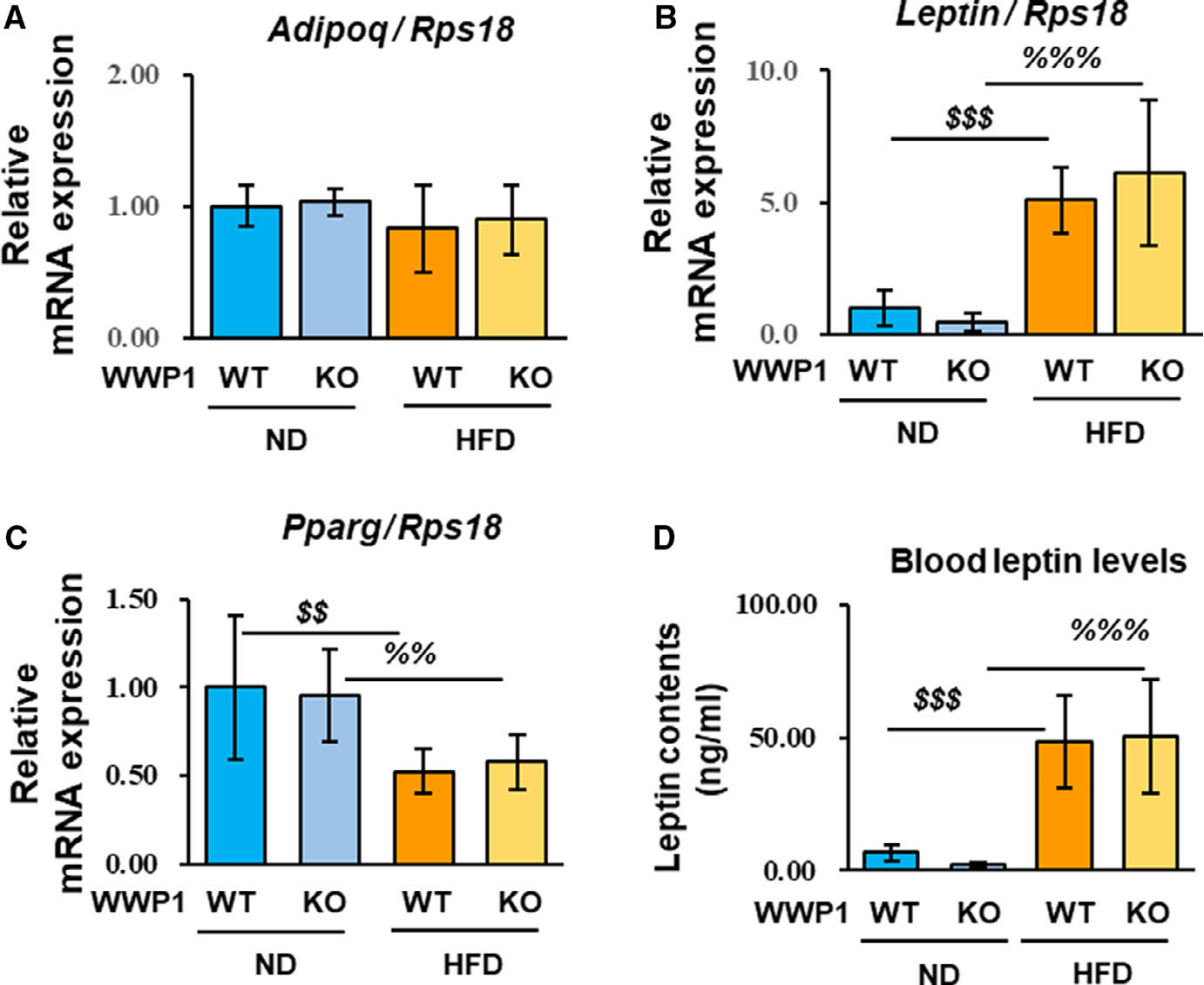
The WWP1 contributes minimally to the regulation of adipokines and *Pparγ* in obese WAT. (A‐C) mRNA levels of adipokines (A) *Adipoq* and (B) *Leptin*, and (C) *Pparγ* in WAT from all groups of mice were measured. (D) Plasma leptin levels from all groups of mice were measured by ELISA. Quantitative data represent mean ± SD (*n* = 6–10 per group). Differences between these values were analyzed using the Tukey–Kramer test [$$, *P* < 0.01, $$$, *P* < 0.001 *Wwp1* WT mice (ND‐fed) vs. *Wwp1* WT mice (HFD‐fed); %%, *P* < 0.01, %%%, *P* < 0.001 *Wwp1* KO mice (ND‐fed) vs. *Wwp1* KO mice (HFD‐fed)].

Increased oxidative stress in adipose tissue is strongly associated with physiological processes and properties of adipocytes such as cell hypertrophy and adipokine imbalance. We examined mRNA levels of adipokines (*Adipoq* (an adipocyte differentiation marker), *Leptin*)*,* and *Pparγ*, and plasma leptin levels. Although there were no changes in *Adipoq* levels, *Leptin* levels were increased, while *Pparγ* levels were decreased in the HFD groups (Fig. 4A–C). Similarly, plasma leptin levels were increased in the HFD groups (Fig. 4D). These factors were unchanged in *Wwp1* KO mice.

## Discussion

In the present study, we showed that WWP1 deficiency reduced the GSH/GSSG ratio because of increased concentration of GSSG in obese WAT. GSH is a tripeptide that contributes to reduction of concentrations of ROS, such as hydrogen peroxide and lipid peroxide 28. An acute increase in ROS rapidly converts GSH to GSSG, resulting in a reduction of GSH/GSSG, without a change in total GSH. In contrast, chronic excess of ROS increases total GSH content, which increases the antioxidant capacity of tissue 29. *Wwp1* KO mice showed normal total GSH content when fed the HFD, indicating that WWP1 may contribute to the acute antioxidative response in WAT during the early stages of obesity. This discrepancy is supported by our previously generated proteomic data, which showed that WWP1 positively regulates the expression of antioxidative proteins 23. We also found that 4‐HNE concentrations were similar in WT and KO mice and in the ND‐ and HFD‐fed groups. As an end‐product of lipid peroxidation, 4‐HNE can be considered a biomarker of chronic or cumulative oxidative stress 30. Given the above findings, it is plausible that HFD‐feeding for 8 weeks induces acute oxidative stress in WAT, but does not fully induce chronic oxidative stress, while WWP1 ameliorates the acute stress. In addition, we measured the levels of antioxidative factors, such as *Txn1/2*, *Prdx1*, *Sod1/2*, and *Catalase* (Fig. S2A–F), although they were unchanged in WAT of *Wwp1* WT and KO mice. In a recent report, we showed that antioxidant factors (e.g., TRX and PRDX) correlated with WWP1 expression according to proteome analysis *in vitro*. However, we were unable to identify any significant changes in protein expression from western blot analysis 23. To our knowledge, the WWP1 substrates that directly contribute to the oxidative stress response have not yet been identified, although TβR1 and Smad2, WWP1 substrates involved in TGFβ (transforming growth factor β) signaling, are reported to be associated with the oxidative stress pathway 11, 31. Therefore, a comprehensive analysis of *Wwp1* WT and KO mice is required to elucidate the mechanisms involved in the WWP1‐related antioxidative response.

As described above, ROS are predominantly generated in mitochondria. CS is an important enzyme in the TCA cycle, and its activity is used as a biomarker of mitochondrial function 32. In general, both CS activity and mitochondrial mass decrease in obesity, and CS activity correlates with mitochondrial mass. However, it has been previously reported that short‐term HFD‐feeding increases CS activity in skeletal muscle 33. This finding suggests that a compensatory upregulation in mitochondrial function may occur to protect against the effects of overnutrition. The findings in skeletal muscle and the present observations regarding oxidative stress in WAT suggest that HFD increases CS activity without increasing mitochondrial mass, while WWP1 deficiency prevents full induction of CS activity in HFD‐fed mice. We also found that WWP1 knockdown reduced maximal respiration in 3T3‐L1 adipocytes (Fig. S3), which was consistent with the *in vivo* data.

Several previous studies have demonstrated a relationship between oxidative stress and insulin signaling. For example, it has been shown that increased mitochondrial ROS production inhibits binding of insulin receptor substrate‐1 to the insulin receptor, resulting in insulin resistance 26.

This implies that lower pAKT levels in the WAT of *Wwp1* KO mice may be attributed to cumulative oxidative stress. However, while plasma insulin levels were also lower, glucose levels were not increased in KO mice. This observation suggests WWP1 deficiency can improve glucose metabolism. Consistent with these findings, GTT and ITT suggested that WWP1 deficiency can improve whole‐body glucose metabolism in obesity. It is reported that WWP1 reduces the activity of phosphatase and tensin homolog (PTEN), which inhibits AKT phosphorylation 34. Therefore, WWP1 deficiency may inhibit PTEN suppression, resulting in reduction of pAkt in *Wwp1* KO WAT. In contrast, according to GTT and ITT, whole‐body glucose metabolism was improved. Because the mice analyzed in this study did not have adipose tissue‐specific KO of *Wwp1*, it cannot be ruled out that WWP1 deletion affected insulin sensitivity in tissues other than WAT (e.g., liver, skeletal muscle, and brain) and secretion of insulin from pancreatic β‐cells.

In this study, the levels of *Leptin* mRNA and plasma leptin were increased in the HFD groups, but *Adipoq* mRNA levels, which are widely known to be downregulated under conditions of obesity, were unchanged. A recent study in mice with HFD‐induced obesity demonstrated that a decrease in leptin levels occurs within 8 weeks of starting the HFD, whereas the decrease of adiponectin occurs over 20 weeks 35. This report supports the validity of our results from mice fed with a HFD for 8 weeks. Increased leptin levels and decreased *Pparγ* levels are generally observed in obesity 36, 37. However, we did not observe an effect on these factors related to *Wwp1* KO obese WAT. Therefore, we concluded that WWP1 or its related antioxidative properties exert minimal effects on adipocytes.

In our previous study, we identified WWP1 as an obesity‐induced factor that positively regulates antioxidative reactions in adipocytes 23. Consistent with these findings, in the present study, we showed that *Wwp1* KO mice exhibit lower obesity‐responsive antioxidant capacity. We also showed that *Wwp1* KO mice have poorer mitochondrial function and insulin signaling capacity. Therefore, we conclude that WWP1 may be involved in maintaining WAT function, including the response to oxidative stress, mitochondrial function, and insulin signaling in obesity. Further investigation of the role of WWP1 in WAT should provide insight into obesity‐related pathology in WAT. In particular, identification of the WWP1 substrates should be prioritized.

## Author contributions

MK and YH conceived the idea, designed experiments, and wrote the manuscript. SH predominantly performed experiments and wrote the manuscript under the leadership of MK and RT. RK, TA, KF, KM, and HW assisted with the experiments. NO and YS contributed to the discussion. YM, YN, TN, and HK supported the experiments.

## Conflict of interest

The authors declare no conflict of interest.

## Supporting information


**Fig. S1** **.** Body mass, tissue masses, and caloric intake of *Wwp1 *WT and KO mice. (A) Protein expression of WWP1. The protein levels of the indicated proteins were determined by immunoblot analysis. The data were normalized to the total protein levels, determined following CBB staining. (B) Body mass and (C) total calorie consumption of C57/BL6 *Wwp1 *WT and KO mice fed a normal diet (ND) or a high‐fat diet (HFD). (D) Mass of WAT in mice fed an ND or an HFD. The quantitative data are means ± SD (*n* = 5–7). The differences between these values were analyzed using the Tukey‐Kramer test. (*$$$*, p<0.001 Wwp1 WT mice (ND‐fed) vs. Wwp1 WT mice (HFDfed); *%%%*, *P* < 0.001 Wwp1 KO mice (ND‐fed) vs. Wwp1 KO mice (HFD‐fed)).
**Fig. S2** **.** WWP1 does not affect of mRNA expression levels of antioxidant factors in WAT. (A–F) mRNA expression levels of antioxidant factors (A) Txn1, (B) Txn2, (C) Prdx1, (D) Sod1, (E) Sod2 and (F) Catalase in WAT from all the groups of mice were measured. Rps18 was used as a housekeeping gene. The quantitative values are means ± SD (n=7–10 per group). Differences between these values were analyzed using the Tukey‐Kramer test [$$, p<0.01, $$$, p<0.001 Wwp1 WT mice (ND‐fed) vs. Wwp1 WT mice (HFD‐fed); %%, p<0.01, %%%, p<0.001 Wwp1 KO mice (ND‐fed) vs. Wwp1 KO mice (HFD‐fed)].
**Fig. S3** **.** WWP1 is required for normal mitochondrial function in mature 3T3‐L1 adipocytes. *In vitro *data from mature 3T3‐L1/shGFP and 3T3‐L1/Wwp1 KD adipocytes are shown. The cells were analyzed using an XF24 analyzer. A time course for the measurement of oxygen consumption rate (OCR) is shown under proton leak conditions, following the addition of oligomycin (1 μm), FCCP (1 μM), and rotenone (1 μM). Values are means ± SD (*n *= 5). Differences between these values were analyzed using Student’s *t*‐test (&, *p*<0.05 &&&, *p*<0.001; 3T3‐L1/shGFP vs. 3T3‐L1/WWP1 KD).

## References

[1] Furukawa S , Fujita T , Shimabukuro M , Iwaki M , Yamada Y , Nakajima Y , Nakayama O , Makishima M , Matsuda M and Shimomura I (2003) Increased oxidative stress in obesity and its impact on metabolic syndrome. J Clin Invest 114, 1752–1761. 10.1172/JCI21625 PMC535065 15599400

[2] Paglialunga S , Ludzki A , Root‐McCaig J and Holloway GP (2015) In adipose tissue, increased mitochondrial emission of reactive oxygen species is important for short‐term high‐fat diet‐induced insulin resistance in mice. Diabetologia 58, 1071–1080. 25754553 10.1007/s00125-015-3531-x

[3] Stadman ER and Levine RL (2003) Free radical‐mediated oxidation of free amino acids and amino acid resides in proteins. Amino Acids 25, 207–218. 14661084 10.1007/s00726-003-0011-2

[4] Richter C , Park JW and Ames BN (1988) Normal oxidative damage to mitochondrial and nuclear DNA is extensive. Proc Natl Acad Sci USA 85, 6465–6467. 3413108 10.1073/pnas.85.17.6465 PMC281993

[5] Rubbo H , Denicola A and Radi R (1994) Peroxynitrite inactivates thiol‐containing enzymes of Trypanosoma cruzi energetic metabolism and inhibits cell respiration. Arch Biochem Biophys 308, 96–102. 8311481 10.1006/abbi.1994.1014

[6] Sakuraba H , Mizukami H , Yagihashi N , Wada R , Hanyu C and Yagihashi S (2002) Reduced beta‐cell mass and expression of oxidative stress‐related DNA damage in the islet of Japanese Type II diabetic patients. Diabetologia 45, 85–96. 11845227 10.1007/s125-002-8248-z

[7] Fernández‐Sánchez A , Madrigal‐Santillán E , Bautista M , Esquivel‐Soto J , Morales‐González A , Esquivel‐Chirino C , Durante‐Montiel I , Sánchez‐Rivera G , Valadez‐Vega C and Morales‐González JA (2011) Inflammation, oxidative stress, and obesity. Int J Mol Sci 12, 3117–3132. 21686173 10.3390/ijms12053117 PMC3116179

[8] Kostapanos MS , Spyrou AT , Tellis CC , Gazi IF , Tselepis AD , Elisaf M and Liberopoulos EN (2011) Ezetimibe treatment lowers indicators of oxidative stress in hypercholesterolemic subjects with high oxidative stress. Lipids 46, 341–348. 21359598 10.1007/s11745-011-3539-z

[9] Manna P and Jain SK (2015) Obesity, oxidative stress, adipose tissue dysfunction, and the associated health risks: causes and therapeutic strategies. Metab Syndr Relat Disord 13, 423–444. 26569333 10.1089/met.2015.0095 PMC4808277

[10] Chattopadhyay M , Khemka VK , Chatterjee G , Ganguly A , Mukhopadhyay S and Chakrabarti S (2015) Enhanced ROS production and oxidative damage in subcutaneous white adipose tissue mitochondria in obese and type 2 diabetes subjects. Mol Cell Biochem 399, 95–103. 25312902 10.1007/s11010-014-2236-7

[11] Seo SR , Lallemand F , Ferrand N , Pessah M , L'Hoste S , Camonis J and Atfi A (2004) The novel E3 ubiquitin ligase Tiul1 associates with TGIF to target Smad2 for degradation. EMBO J 23, 3780–3792. 15359284 10.1038/sj.emboj.7600398 PMC522797

[12] Zhi X and Chen C (2012) WWP1: a versatile ubiquitin E3 ligase in signaling and diseases. Cell Mol Life Sci 69, 1425–1434. 22051607 10.1007/s00018-011-0871-7 PMC11114891

[13] Chen C , Zhou Z , Ross JS , Zhou W and Dong JT (2007) The amplified WWP1 gene is a potential molecular target in breast cancer. Int J Cancer 121, 80–87. 17330240 10.1002/ijc.22653

[14] Imamura M , Nakamura A , Mannen H and Takeda S (2016) Characterization of WWP1 protein expression in skeletal muscle of muscular dystrophy chickens. J Biochem 159, 171–179. 26314333 10.1093/jb/mvv084 PMC4892772

[15] Hirata Y , Nomura K , Senga Y , Okada Y , Kobayashi K , Okamoto S , Minokoshi Y , Imamura M , Takeda S , Hosooka T *et al* (2019) Hyperglycemia induces skeletal muscle atrophy via a WWP1/KLF15 axis. J.C.I. Insight 4, e124952. 10.1172/jci.insight.124952 PMC6478420 30830866

[16] Ambrozkiewicz MC , Schwark M , Kishimoto‐Suga M , Borisova E , Hori K , Salazar‐Lázaro A , Rusanova A , Altas B , Piepkorn L , Bessa P *et al* (2018) Polarity acquisition in cortical neurons is driven by synergistic action of Sox9‐regulated Wwp1 and Wwp2 E3 ubiquitin ligases and intronic miR‐140. Neuron 100, 1097–1115. 30392800 10.1016/j.neuron.2018.10.008

[17] Conkright MD , Wani MA and Lingrel JB (2001) Lung Krüppel‐like factor contains an autoinhibitory domain that regulates its transcriptional activation by binding WWP1, an E3 ubiquitin ligase. J Biol Chem 276, 29299–29306. 11375995 10.1074/jbc.M103670200

[18] Zhang X , Srinivasan SV and Lingrel JB (2004) WWP1‐dependent ubiquitination and degradation of the lung Kruppel‐like factor KLF2. Biochem Biophys Res Commun 316, 139–148. 15003522 10.1016/j.bbrc.2004.02.033

[19] Chen C , Sun X , Guo P , Dong XY , Sethi P , Cheng X , Zhou J , Ling J , Simons JW , Lingrel JB *et al* (2005) Human Krüppel‐like factor 5 is a target of the E3 ubiquitin ligase WWP1 for proteolysis in epithelial cells. J Biol Chem 280, 41553–41561. 16223724 10.1074/jbc.M506183200

[20] Eastman SW , Yassaee M and Bieniasz PD (2009) A role for ubiquitin ligases and Spartin/SPG20 in lipid droplet turnover. J Cell Biol 184, 881–894. 19307600 10.1083/jcb.200808041 PMC2699154

[21] Edwards TL , Clowes VE , Tsang HT , Connell JW , Sanderson CM , Luzio JP and Reid E (2009) Endogenous spartin (SPG20) is recruited to endosomes and lipid droplets and interacts with the ubiquitin E3 ligases AIP4 and AIP5. Biochem J 423, 31–39. 19580544 10.1042/BJ20082398 PMC2762690

[22] Oishi Y , Manabe I , Tobe K , Ohsugi M , Kubota T , Fujiu K , Maemura K , Kubota N , Kadowaki T and Nagai R (2008) SUMOylation of Krüppel‐like transcription factor 5 acts as a molecular switch in transcriptional programs of lipid metabolism involving PPAR‐delta. Nat Med 14, 656–666. 18500350 10.1038/nm1756

[23] Kobayashi M , Hoshino S , Abe T , Okita N , Tagawa R , Nagai W , Konno R , Suzuki Y , Furuya K , Ishikawa N *et al* (2019) Identification of WWP1 as an obesity‐associated E3 ubiquitin ligase with a protective role against oxidative stress in adipocytes. Biochem Biophys Res Commun 508, 117–122. 30471861 10.1016/j.bbrc.2018.11.127

[24] Okita N , Hayashida Y , Kojima Y , Fukushima M , Yuguchi K , Mikami K , Yamauchi A , Watanabe K , Noguchi M , Nakamura M *et al* (2012) Differential responses of white adipose tissue and brown adipose tissue to caloric restriction in rats. Mech Ageing Dev 133, 255–266. 22414572 10.1016/j.mad.2012.02.003

[25] Rahman I , Kode A and Biswas SK (2006) Assay for quantitative determination of glutathione and glutathione disulfide levels using enzymatic recycling method. Nat Protoc 1, 3159–3165. 17406579 10.1038/nprot.2006.378

[26] Imoto K , Kukidome D , Nishikawa T , Matsuhisa T , Sonoda K , Fujisawa K , Yano M , Motoshima H , Taguchi T , Tsuruzoe K *et al* (2006) Impact of mitochondrial reactive oxygen species and apoptosis signal‐regulating kinase 1 on insulin signaling. Diabetes 55, 1197–1204. 16644673 10.2337/db05-1187

[27] Mlinar B and Marc J (2011) New insights into adipose tissue dysfunction in insulin resistance. Clin Chem Lab Med 49, 1925–1935. 21892913 10.1515/CCLM.2011.697

[28] Wu G , Fang YZ , Yang S , Lupton JR and Turner ND (2004) Glutathione metabolism and its implications for health. J Nutr 134, 489–492. 14988435 10.1093/jn/134.3.489

[29] Asensi M , Sastre J , Pallardo FV , Lloret A , Lehner M , Garcia‐de‐la Asuncion J and Viña J (1999) Ratio of reduced to oxidized glutathione as indicator of oxidative stress status and DNA damage. Methods Enzymol 299, 267–276. 9916205 10.1016/s0076-6879(99)99026-2

[30] Kim CS , Lee SC , Kim YM , Kim BS , Choi HS , Kawada T , Kwon BS and Yu R (2008) Visceral fat accumulation induced by a high‐fat diet causes the atrophy of mesenteric lymph nodes in obese mice. Obesity 16, 1261–1269. 18369345 10.1038/oby.2008.55

[31] Komuro A , Imamura T , Saitoh M , Yoshida Y , Yamori T , Miyazono K and Miyazawa K (2004) Negative regulation of transforming growth factor‐beta (TGF‐beta) signaling by WW domain‐containing protein 1 (WWP1). Oncogene 23, 6914–23. 15221015 10.1038/sj.onc.1207885

[32] Wiegand G and Remington SJ (1986) Citrate synthase: structure, control, and mechanism. Annu Rev Biophys Chem 15, 97–117. 10.1146/annurev.bb.15.060186.000525 3013232

[33] Li X , Higashida K , Kawamura T and Higuchi M (2016) Alternate‐day high‐fat diet induces an increase in mitochondrial enzyme activities and protein content in rat skeletal muscle. Nutrients. 8, 203. 27058555 10.3390/nu8040203 PMC4848672

[34] Lee YR , Chen M , Lee JD , Zhang J , Lin SY , Fu TM , Chen H , Ishikawa T , Chiang SY , Katon J *et al* (2019) Reactivation of PTEN tumor suppressor for cancer treatment through inhibition of a MYC‐WWP1 inhibitory pathway. Science 364, eaau0159. 31097636 10.1126/science.aau0159 PMC7081834

[35] Kwon EY , Shin SK , Cho YY , Jung UJ , Kim E , Park T , Park JH , Yun JW , McGregor RA , Park YB *et al* (2012) Time‐course microarrays reveal early activation of the immune transcriptome and adipokine dysregulation leads to fibrosis in visceral adipose depots during diet‐induced obesity. BMC Genom 13, 450. 10.1186/1471-2164-13-450 PMC3447724 22947075

[36] Lodhi IJ , Yin L , Jensen‐Urstad AP , Funai K , Coleman T , Baird JH , El Ramahi MK , Razani B , Song H , Fu‐Hsu F *et al* (2012) Inhibiting adipose tissue lipogenesis reprograms thermogenesis and PPARγ activation to decrease diet‐induced obesity. Cell Metab 16, 189–201. 22863804 10.1016/j.cmet.2012.06.013 PMC3467338

[37] Step SE , Lim HW , Marinis JM , Prokesch A , Steger DJ , You SH , Won KJ and Lazar MA (2014) Anti‐diabetic rosiglitazone remodels the adipocyte transcriptome by redistributing transcription to PPARγ‐driven enhancers. Genes Dev 28, 1018–1028. 24788520 10.1101/gad.237628.114 PMC4018489

