# Supplementary material for: WWP1 knockout in mice exacerbates obesity‐related phenotypes in white adipose tissue but improves whole‐body glucose metabolism
Source: FEBS Open Bio. 2020 Feb 3;10(3):306–15. doi: 10.1002/2211-5463.12795 (PMC7050250; doi:10.1002/2211-5463.12795)
Supplement: Supplementary file 1 — Fig. S1 . Body mass, tissue masses, and caloric intake of Wwp1 WT and KO mice. (A) Protein expression of WWP1. The protein levels of the indicated proteins were determined by immunoblot analysis. The data were normalized to the total protein levels, determined following CBB staining. (B) Body mass and (C) total calorie consumption of C57/BL6 Wwp1 WT and KO mice fed a normal diet (ND) or a high‐fat diet (HFD). (D) Mass of WAT in mice fed an ND or an HFD. The quantitative data are means ± SD (n = 5–7). The differences between these values were analyzed using the Tukey‐Kramer test. ($$$, p<0.001 Wwp1 WT mice (ND‐fed) vs. Wwp1 WT mice (HFDfed); %%%, P < 0.001 Wwp1 KO mice (ND‐fed) vs. Wwp1 KO mice (HFD‐fed)). Fig. S2 . WWP1 does not affect of mRNA expression levels of antioxidant factors in WAT. (A–F) mRNA expression levels of antioxidant factors (A) Txn1, (B) Txn2, (C) Prdx1, (D) Sod1, (E) Sod2 and (F) Catalase in WAT from all the groups of mice were measured. Rps18 was used as a housekeeping gene. The quantitative values are means ± SD (n=7–10 per group). Differences between these values were analyzed using the Tukey‐Kramer test [$$, p<0.01, $$$, p<0.001 Wwp1 WT mice (ND‐fed) vs. Wwp1 WT mice (HFD‐fed); %%, p<0.01, %%%, p<0.001 Wwp1 KO mice (ND‐fed) vs. Wwp1 KO mice (HFD‐fed)]. Fig. S3 . WWP1 is required for normal mitochondrial function in mature 3T3‐L1 adipocytes. In vitro data from mature 3T3‐L1/shGFP and 3T3‐L1/Wwp1 KD adipocytes are shown. The cells were analyzed using an XF24 analyzer. A time course for the measurement of oxygen consumption rate (OCR) is shown under proton leak conditions, following the addition of oligomycin (1 μm), FCCP (1 μM), and rotenone (1 μM). Values are means ± SD (n = 5). Differences between these values were analyzed using Student’s t‐test (&, p<0.05 &&&, p<0.001; 3T3‐L1/shGFP vs. 3T3‐L1/WWP1 KD). [file FEB4-10-306-s001.pdf]

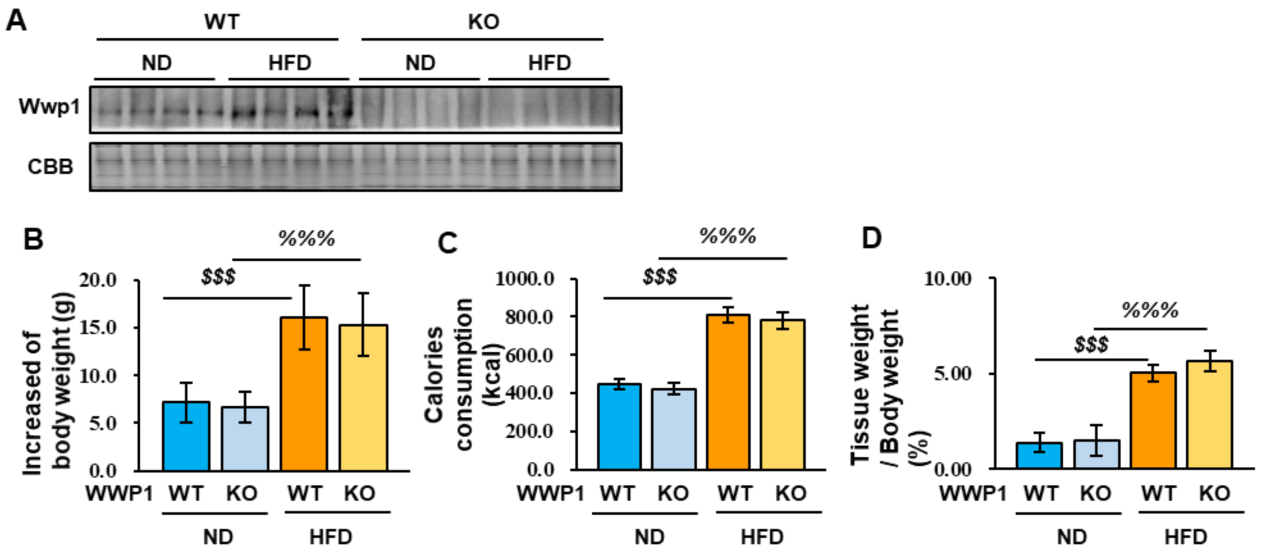

Fig. S1 Body mass, tissue masses, and caloric intake of *Wwp1* WT and KO mice
(A) Protein expression of WWP1. The protein levels of the indicated proteins were determined by immunoblot analysis. The data were normalized to the total protein levels, determined following CBB staining. (B) Body mass and (C) total calorie consumption of C57/BL6 *Wwp1* WT and KO mice fed a normal diet (ND) or a high-fat diet (HFD). (D) Mass of WAT in mice fed an ND or an HFD. The quantitative data are means ±SD (n=5–7). The differences between these values were analyzed using the Tukey-Kramer test. (*$$$*, p<0.001 Wwp1 WT mice (ND-fed) vs. Wwp1 WT mice (HFD-fed); *%%%*, p<0.001 Wwp1 KO mice (ND-fed) vs. Wwp1 KO mice (HFD-fed)).

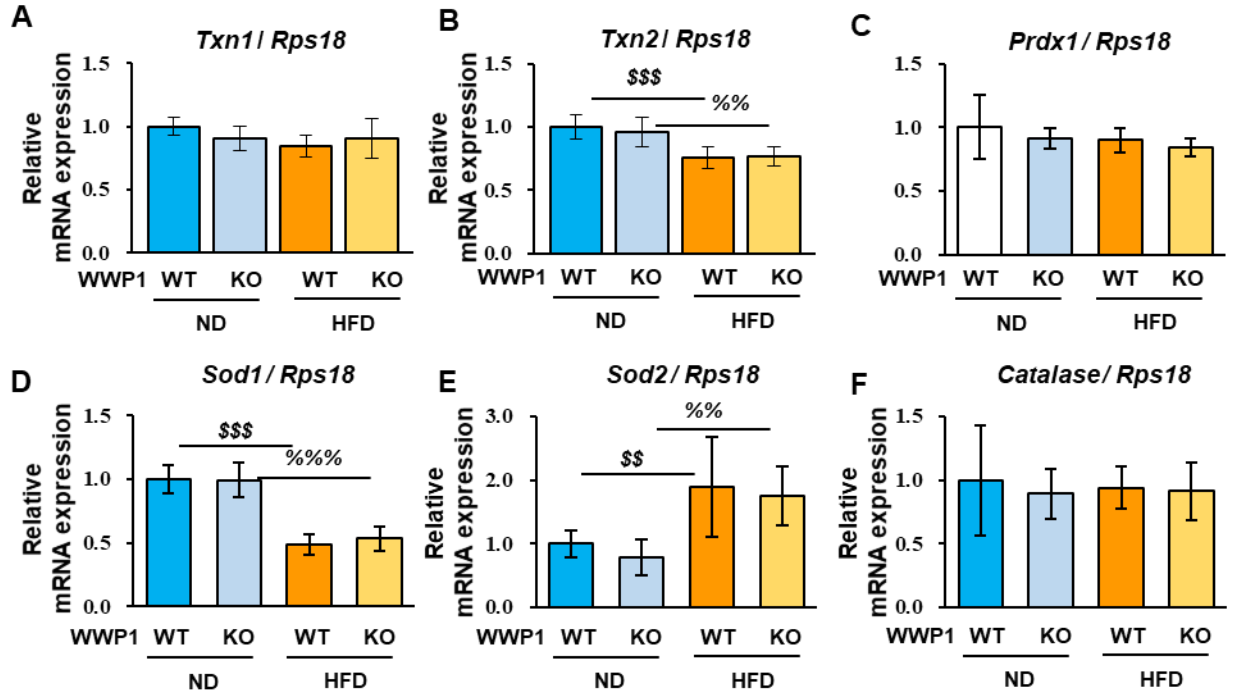

Fig. S2 WWP1 does not affect of mRNA expression levels of antioxidant factors in WAT.
(A-F) mRNA expression levels of antioxidant factors (A) Txn1, (B) Txn2, (C) Prdx1, (D) Sod1, (E) Sod2 and (F) Catalase in WAT from all the groups of mice were measured. Rps18 was used as a housekeeping gene. The quantitative values are means ± SD (n=7–10 per group). Differences between these values were analyzed using the Tukey-Kramer test ($$, p<0.01, $$$, p<0.001 Wwp1 WT mice (ND-fed) vs. Wwp1 WT mice (HFD-fed); %%, p<0.01, %%%, p<0.001 Wwp1 KO mice (ND-fed) vs. Wwp1 KO mice (HFD-fed)).

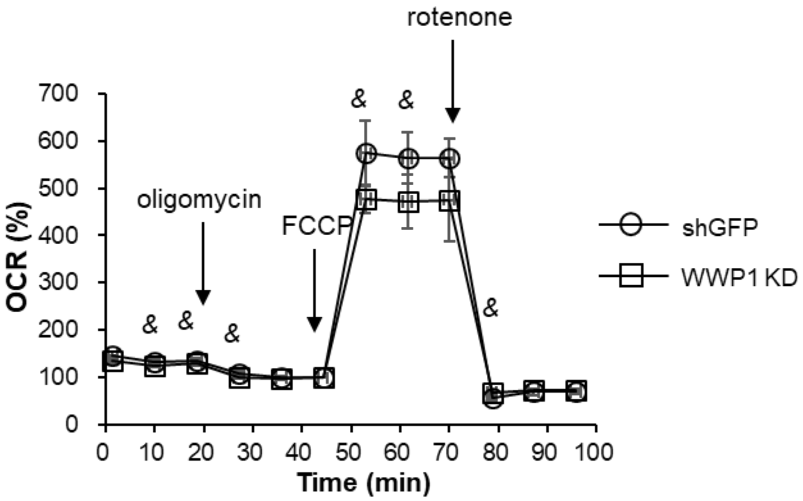

Fig. S3 WWP1 is required for normal mitochondrial function in mature 3T3-L1 adipocytes
In vitro data from mature 3T3-L1 / shGFP and 3T3-L1 / Wwp1 KD adipocytes are shown. The cells were analyzed using an XF24 analyzer. A time course for the measurement of oxygen consumption rate (OCR) is shown under proton leak conditions, following the addition of oligomycin (1 μM), FCCP (1 μM), and rotenone (1 μM). Values are means ± SD (n = 5). Differences between these values were analyzed using Student's *t*-test (&, $p<0.05$ &&&, $p<0.001$; 3T3-L1 / shGFP *vs*. 3T3-L1 / WWP1 KD).
